# Perceived chronic social adversity and cyberbullying perpetration among adolescents: the mediating role of rumination and moderating role of mindfulness

**DOI:** 10.3389/fpsyg.2024.1376347

**Published:** 2024-06-06

**Authors:** Rui Chen, Yang Hu, Hui-fen Shi, Yong Fang, Cui-ying Fan

**Affiliations:** ^1^Key Laboratory of Adolescent Cyberpsychology and Behavior (CCNU), Ministry of Education, Wuhan, China; ^2^School of Psychology, Central China Normal University, Wuhan, China; ^3^Key Laboratory of Human Development and Mental Health of Hubei Province, Wuhan, China; ^4^School of Medical Humanities, Hubei University of Chinese Medicine, Wuhan, China; ^5^Hubei Shizhen Laboratory, Wuhan, China; ^6^School of Preschool Education, Hubei Preschool Teachers College, Wuhan, China; ^7^School of Nursing, Hubei University of Chinese Medicine, Wuhan, China

**Keywords:** perceived chronic social adversity, rumination, mindfulness, cyberbullying perpetration, adolescents

## Abstract

**Backgrounds:**

The prevalence of cyberbullying has brought about many adverse effects on adolescents’ mental health. Although current studies have shown that perceived chronic social adversity (PCSA) is closely related to cyberbullying perpetration among adolescents, the underlying mechanism of the relationship between the two remains relatively unclear. This study investigated the association of PCSA, rumination, mindfulness, and cyberbullying perpetration among adolescents, building upon the general strain theory, the general aggressive model, and the limited resource of self-control theory.

**Methods:**

A sample of 477 Chinese high school students (*M*_age_ = 15.84 years, *SD*_age_ = 0.67, 49.69% female) completed the Perceived Chronic Social Adversity Questionnaire, the Ruminative Responses Scale, the Child and Adolescent Mindfulness Measure, and the cyberbullying subscale of the Revised Cyber Bullying Inventory. The current study constructed a moderated mediation model to examine the relationship between PCSA and cyberbullying perpetration among adolescents and assessed the mediating role of rumination and the moderating role of mindfulness.

**Results:**

The results revealed a significant positive correlation between PCSA and cyberbullying perpetration. Rumination mediated the relationship between PCSA and cyberbullying perpetration, whereas mindfulness moderated the latter half of the mediation pathway. Specifically, compared to adolescents with higher mindfulness, the association between rumination and cyberbullying perpetration is greater for adolescents with lower mindfulness.

**Conclusion:**

The results further deepen our understanding of the mechanisms linking subjective perception of negative life events and cyberbullying perpetration among adolescents from the interaction of multiple factors, thus providing a basis for future interventions to encourage adolescents to properly cope with social adversity and promote positive mental health to reduce the risk of cyberbullying.

## 1 Introduction

The way that people establish relationships and carry out daily communication has changed dramatically in recent years with the rapid development and proliferation of the Internet. Simultaneously, an unintended consequence of the increasing use of the Internet is cyberbullying. Cyberbullying refers to “An aggressive, intentional act carried out by a group or individual, using electronic forms of contact, repeatedly and over time against a victim who cannot easily defend him or herself” (Smith et al., 2008), with the properties of interpersonal anonymity, temporal asynchrony, spatial transcendence, and self-disinhibition (Zhou and Liu, 2016). These properties minimize the punishment consequences for cyberbullying perpetrators and make cyberbullying more likely to occur (Watts et al., 2017). For instance, cyberbullying prevalence has ranged from 17 to 38% among Chinese adolescents (Chu et al., 2018; Yang et al., 2018). Previous studies have shown that cyberbullying may lead to a range of negative outcomes, including loneliness, depression, anxiety, drug abuse, and suicidal ideation (Brewer and Kerslake, 2015; Guo, 2016; Rodelli et al., 2018; Gao et al., 2023). Besides, the prevalence of cyberbullying and its negative outcomes among adolescents is still rising (Chu et al., 2019; Fan et al., 2019). Hence, it is of great realistic significance to explore the mechanism of cyberbullying occurrence among adolescents.

Prior studies have revealed that the influencing factors of cyberbullying mainly include environmental factors and individual factors (Ding et al., 2020). Environmental factors mainly include stressors (Islam et al., 2022), family socioeconomic status (Yang et al., 2023), parenting style (Hinduja and Patchin, 2022), and school atmosphere (Ding et al., 2020). Individual factors include personality characteristics (Zhang et al., 2020), emotional state (Kowalski et al., 2014), self-related cognition, social competence, and academic performance (Ding et al., 2020). However, the existing literature primarily addresses the roles of environmental factors and individual factors from a binary perspective (Guo, 2016; Gao et al., 2023), making it difficult to effectively explain the differences in cyberbullying among different groups of adolescents. According to the general aggressive model (Anderson and Bushman, 2002), aggressive behaviors such as cyberbullying perpetration are generated by the interaction of individual factors and environmental factors (DeWall et al., 2011; Fan et al., 2018). As an environmental factor, negative life events involve a series of the most common unpleasant events in the course of individuals’ lives that hinder the normal development of adolescents’ cognition, emotional and behavioral patterns, for instance, obvious or obscure social exclusion or alienation, being overly controlled, and failure in social competition (Zhang JQ et al., 2017). It has a significant association with an individual’s aggressive behaviors (Huang et al., 2017; Shao et al., 2018). However, the perception of negative life events by individuals may lead to different behavioral outcomes owing to the different interactions between individual factors and environmental factors (Jin et al., 2020). This means that different adolescents may face different risks of cyberbullying perpetration due to perceptual differences when facing the same negative life event. Thus, understanding the effects of interaction such as subjective perception of negative life events on cyberbullying perpetration is especially pressing. However, the relationship between subjective perception of negative life events and cyberbullying perpetration among adolescents remains unclear, so it is valuable to explore the underlying mechanisms of them and provide efficient practical guidance for decreasing the prevalence of cyberbullying incidents.

Perceived chronic social adversity (PCSA) refers to an individual’s subjective perception of persistent or recurring negative life events (Zhang JQ et al., 2017). Although these events are not life-and-safety threatening events, cumulative experiences of them will induce chronic psychological distress (Zhang J et al., 2017). Based on the general strain theory (Agnew, 1992), when individuals with high PCSA receive psychological distress generated by social adversity, they are more likely to engage in aggressive behaviors, to restore the balance in cognitive, emotional, and behavioral aspects (Jin et al., 2016). Previous studies have shown that PCSA is associated with an individual’s anxiety, depression, anger, and aggressive behaviors (Zhang JQ et al., 2017; Jin et al., 2020; Liu et al., 2022). Compared with psychological trauma, PCSA is characterized by repetition and continuity, which is more likely to reflect the universality and long-term cumulative effect of negative life events (Zhang J et al., 2017; Ding et al., 2018). The cumulative effect and its negative impact are more pronounced in adolescents (Shao et al., 2018). This is because high-order cognitive processes about negative life events, such as subjective evaluation and attribution, will stimulate adolescents’ immature thinking and unstable emotions, and are more likely to trigger their impulsive physiological and behavioral responses related to aggressive tendencies (Berkowitz, 2012). Aggressive behavior is often manifested in the form of cyberbullying in adolescents (Chu et al., 2019). Empirical evidence has indicated that high PCSA is associated with increased cyberbullying perpetration (Liu, 2023). Specifically, for adolescents, perceiving adverse events such as being overcontrolled by parents is significantly positively correlated with cyberbullying perpetration (Li, 2020). Meanwhile, those marked by exclusion and other emotional trauma are more likely to bully others in cyberspace (Kircaburun et al., 2020; Wang et al., 2020). Therefore, we consider that PCSA is positively related to cyberbullying perpetration among adolescents, this study formulated the following hypothesis:

*H1*: PCSA is significantly and positively associated with cyberbullying perpetration among adolescents.

PCSA, as a form of individuals’ core characteristics facing negative life events (Zhang J et al., 2017), plays a crucial role in guiding aggressive behaviors (Jin et al., 2020). The general aggressive model suggests that there is not only a direct association between individuals’ core characteristics and cyberbullying perpetration, but also an indirect one through the mediation of maladaptive cognitive strategy (Anderson and Bushman, 2002; Pedersen et al., 2011; Ruddle et al., 2017; Kokkinos and Antoniadou, 2019). Studies have shown that, as a typical maladaptive cognitive strategy, rumination is a key factor that links external risk factors and individuals’ aggressive behaviors (Eisenlohr-Moul et al., 2016; García-Sancho et al., 2016). Rumination involves repetitively thinking about symptoms, causes, and consequences of social adversity, and long-term activation of negative affect without engagement in active coping to alleviate dysphoric mood (Nolen-Hoeksema et al., 2008). Additional literature confirmed a significant positive correlation between PCSA and rumination (Jin et al., 2020). This is because PCSA is regarded as a persistent, non-life-threatening social trauma, which may make an individual more sensitive to negative information (Baugerud et al., 2016), and undermine the individual’s previous assumptions, goals, and beliefs about themselves, then initiating psychological distress and cognitive rumination processes in an attempt to seek new meaning for social adversity (Calhoun and Tedeschi, 2014). The Stimulation-Cognition-Emotion Model theory also considers PCSA as one of the main indicators for evaluating negative cognition and emotion (Liu and Zhong, 2023), especially individuals whose PCSA reaches the load limit of negative cognition are more likely to engage in rumination (Michl et al., 2013; Liu and Wang, 2017).

Additionally, Selby et al. (2009) note that the higher the level of rumination, the more negative emotions such as anxiety and depression, which in turn enhance rumination and form a vicious cycle, known as the “emotional cascade.” Adolescents lack adequate emotion-regulatory strategies to interrupt the emotional cascade process, those with higher rumination tend to prefer aggressive behaviors like cyberbullying perpetration to temporarily shift their attention from rumination to others when feeling the sustained pressure caused by negative emotions (Jang et al., 2014). Prior studies have shown that rumination is positively associated with individuals’ aggressive behaviors (Eisenlohr-Moul et al., 2016; García-Sancho et al., 2016; Jin et al., 2020), and cyberbullying is a typical sub-category of aggressive behavior in cyberspace (Smith et al., 2008). Accordingly, literature has found a significant positive correlation between angry rumination and cyberbullying perpetration among adolescents (Camacho et al., 2021; Gao et al., 2021; Yang J et al., 2021). Luo et al. (2023) also confirmed that the tendency to ruminate has a significant association with an individual’s cyberbullying perpetration. Moreover, Parris et al. (2022) suggested that high school students who engage in social media rumination may be more likely to be cyberbullying perpetrators. Thus, a positive correlation may exist between rumination and cyberbullying perpetration among adolescents. Drawing on the general aggressive model and relevant empirical studies, this study formulated the following hypothesis:

*H2*: Rumination mediates the relationship between PCSA and cyberbullying perpetration among adolescents.

When exploring the relationship of PCSA and rumination with cyberbullying perpetration, it is essential to consider the role of mindfulness. While an adolescent’s PCSA level can impact bullying behaviors in cyberspace through the mediation of rumination, not all adolescents with high rumination necessarily engage in cyberbullying perpetration. Some protective factors may buffer the effects of rumination on cyberbullying perpetration. Watkins and Brown (2002) proposed that individuals with high rumination focus their attention on negative life experiences, leading to impaired self-control functions and uncontrol of aggressive behaviors. According to the limited resource of self-control theory, successful self-control relies on the source of self-control, which is limited and easily consumed within a certain period (Baumeister et al., 2007). Previous studies also confirmed that the predictive effect of rumination on adolescents’ externalizing depends on the consumption of self-control resources through the vicious cycle of negative emotions (White and Turner, 2014; Zhang et al., 2022). In this context, mindfulness is a vital protective factor for individuals’ behavior control (Lindsay and Creswell, 2017). It refers to the conscious, non-judgmental, open, and receptive attitude about the present moment, which is beneficial for restoring self-control resources and enhancing self-control ability (Brown et al., 2007). This is because the individuals with higher mindfulness focus more on attention itself rather than the goal of attention (Ramel et al., 2004), and they are no longer eager to alleviate and eliminate uncertainty and negative experiences related to themselves, thereby improving their cognitive resource conversion efficiency and executive control function (Kraemer et al., 2016; Xiong et al., 2023). When people have sufficient self-control resources, they carefully consider alternative explanations about social adversity, the negative immersive thinking and aggression might not happen (Allen et al., 2018). Therefore, as a stable protective factor, mindfulness is an important mental resource for individuals with deep rumination (Bowlin and Baer, 2012; Bajaj and Pande, 2016), which might be able to moderate the effect of rumination on adolescents’ cyberbullying perpetration. That is, individuals with different levels of mindfulness could choose whether to commit cyberbullying perpetration differently when faced with the same situation or emotional state (Xiong et al., 2023).

Drawing from the limited resource of self-control theory and pertinent empirical literature, we hypothesize that, higher mindfulness adolescents could alleviate the negative effect of rumination on their social adaptation and reduce cyberbullying perpetration, and lower mindfulness adolescents are less likely to reduce negative emotions through active adjusting cognitive strategies, and the negative effect of rumination on cyberbullying perpetration will be more prominent. Therefore, this study formulated the following hypothesis:

*H3*: Mindfulness moderates the relationship between rumination and cyberbullying perpetration among adolescents.

Based on the above, it is particularly important to find out the influencing factors and mechanisms of cyberbullying perpetration among adolescents and to form scientific prevention and intervention programs on this basis. In the present study, building upon the general strain theory, we investigated the connection between PCSA and cyberbullying perpetration among adolescents, thereby expanding this theory to encompass the cyberbullying domain. To date, few studies have examined the underlying mechanisms linking subjective perception of negative life events and cyberbullying from the habitual characteristics of cognitive strategy standpoint, such as rumination. Grounded in the general aggressive model, the current study explores the applicability of this model within cyberbullying perpetration settings, positing that PCSA may not only directly stimulate cyberbullying perpetration but may also do so indirectly via the mediation of rumination. Moreover, there is a dearth of research concerning the moderating mechanisms between rumination and cyberbullying perpetration among adolescents. According to the limited resource of self-control theory, the level of mindfulness may determine whether an adolescent ultimately engages in cyberbullying perpetration, potentially offering vital insights for future effective intervention strategies. As such, this study offers an in-depth investigation of the moderating role of mindfulness within the relationship between rumination and cyberbullying perpetration among adolescents, which can not only expand the study of the factors influencing cyberbullying perpetration at the theoretical level but also provide evidence for intervention of cyberbullying perpetration among adolescents at the practical level.

**Figure 1:**
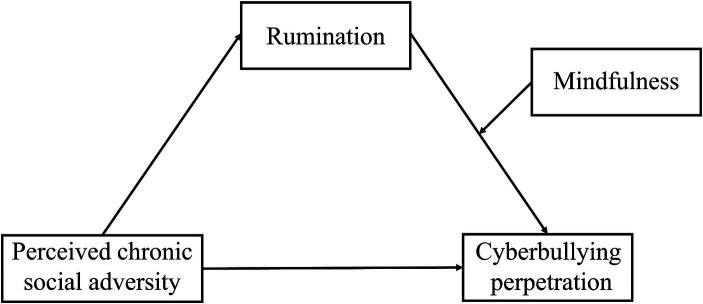
The hypothesized moderated mediation model.

Overall, drawing from both theoretical and empirical perspectives, this study constructed a moderated mediation model described as follows (see Figure 1): (1) PCSA may significantly and positively associate with cyberbullying perpetration; (2) PCSA may associate with cyberbullying perpetration through the mediating effect of rumination; (3) Mindfulness may serve as a moderator between rumination and cyberbullying perpetration among adolescents.

## 2 Materials and methods

### 2.1 Participants

Cluster sampling was adopted to recruit 500 high school students with experience on the Internet to participate in this study. All the participants were recruited from three high schools in Wuhan China. This school age group was considered at elevated risk in social, emotional, academic difficulties, and cyberbullying (Chu et al., 2019; Fan et al., 2019; Shutzman and Gershy, 2023). Before the investigation, all participants were told that the research was being conducted anonymously and that their information would remain confidential. They were informed of the requirements of this survey by using standard instructions, emphasizing the authenticity, independence, and integrity of all answers. All the questionnaires were conducted in the form of paper-and-pencil in different classrooms taking a class as a unit in 45 min. All participants completed an informed consent form prior to completing the questionnaire, and this study was reviewed and approved by the Ethical Committee for Scientific Research of correspondence author. After getting rid of the invalid questionnaires (questionnaires with lots of blanks or repeated answers), data from 477 participants were retained, and the valid response rate was 95.40%. The effective sample included 240 (50.31%) boys and 237 (49.69%) girls. The mean age of participants was 15.84 years old (*SD* = 0.67), with an age range of 14–17 years old. Moreover, a total of 248 (51.99%) first-grade students and 229 (48.01%) second-grade students participated in this survey, the third-grade students did not participate in this survey because of the pressure of college entrance exams.

### 2.2 Measures

#### 2.2.1 Perceived chronic social adversity

This variable was measured by the Chinese version of Perceived Chronic Social Adversity Questionnaire (PCSAQ; Zhang J et al., 2017). This questionnaire comprises 28 items that assess three factors related to PCSA including obvious or obscure social exclusion or alienation, being overly controlled, as well as weakness in social competition (e.g., “Always being rejected”). Participants responded on a five-point scale (1 = totally disagree, 5 = totally agree). Higher scores indicate a greater perceived chronic social adversity. In this study, its Cronbach’s alpha value was 0.82.

#### 2.2.2 Rumination

Rumination was measured by the Chinese version (Han and Yang, 2009) of the Ruminative Responses Scale (Nolen-Hoeksema, 1991). This scale assesses three factors of rumination, including symptom rumination, brooding, and reflective pondering, which consists of 22 items (e.g., “I often think about my shortcomings, failures, errors, and mistakes”) that are scored on a four-point scale (1 = never, 4 = always). Higher scores reflect a higher tendency to the ruminative mode of thinking. In this study, its Cronbach’s alpha value was 0.80.

#### 2.2.3 Mindfulness

This variable was examined using the Chinese version (Liu et al., 2017) of the Child and Adolescent Mindfulness Measure (Greco et al., 2011). It is a single-dimension measurement with 10 items that are answered on a scale from 1 (never) to 5 (always). The items assess two parts of mindfulness, including the lack of awareness and judgment about the present, and the lack of acceptance of thoughts and feelings (e.g., “I do not care about the ideas I do not like”). All items were reverse-scored and the higher scores indicate higher levels of mindfulness in daily life. In this study, its Cronbach’s alpha value was 0.78.

#### 2.2.4 Cyberbullying perpetration

Cyberbullying perpetration was evaluated by the Chinese version (Chu and Fan, 2017) of the cyberbullying subscale of the Revised Cyber Bullying Inventory developed by Topcu and Erdur-Baker (2010). This subscale contains 14 items with each item assessing how frequently adolescents engaged in cyberbullying perpetration in the past 6 months (e.g., “published false photos or information online to defame someone”). Participants responded on a four-point scale (1 = never, 4 = more than three times). Higher scores indicated more frequent cyberbullying perpetration. In this study, its Cronbach’s alpha value was 0.96.

### 2.3 Data analysis and control variables

SPSS 25.0 and the PROCESS macro developed by Hayes (2013) were used for statistical analysis. Firstly, we calculated descriptive statistics and correlational analyses for the main variables. Secondly, the mediation effects (model 4) and moderated mediation effects (model 14) were investigated. The bootstrapping method was used to obtain 95% confidence intervals (95% CI) with 5,000 re-samples for the model. Finally, all the potential significant interaction effects were decomposed by simple slope tests.

Gender and age were included as control variables in our statistical analysis, as previous literature found that they were closely related to the observed variables (Pearson et al., 2011; Yang XJ et al., 2021; Gao et al., 2023; Xiong et al., 2023).

## 3 Results

### 3.1 Common method bias analysis

The data of the present study were all from self-report questionnaires. Therefore, Harman’s single-factor test was used to examine common method bias (Podsakoff et al., 2003), the results indicated that there were 14 factors with eigenvalues higher than 1. The first factor accounted for 25.70% of the variance so common-method variance was not an issue in this study.

### 3.2 Preliminary analysis

Table 1 presented the means, standard deviations, and correlations for all core observed variables. As hypothesized, PCSA was positively correlated with rumination and cyberbullying perpetration, and negatively correlated with mindfulness. Rumination was positively correlated with cyberbullying perpetration, and negatively correlated with mindfulness. Cyberbullying perpetration was negatively correlated with mindfulness. Gender was positively correlated with PCSA and negatively correlated with mindfulness. Age was negatively correlated with PCSA. Whereas, gender and age showed no significant correlation with other core observed variables.

**Table 1:** Descriptive statistics and correlations between variables.

Variables	*M*	*SD*	1	2	3	4	5	6
1. Gender	—	—	—					
2. Age	15.84	0.67	−0.06	1				
3. PCSA	2.20	0.89	0.13^***^	−0.11^**^	1			
4. Rumination	2.45	0.52	−0.03	−0.07	0.44^***^	1		
5. Mindfulness	3.51	0.69	−0.10^***^	0.05	−0.37^***^	−0.23^***^	1	
6. CP	1.12	0.41	0.09	0.03	0.26^***^	0.20^***^	−0.15^***^	1

*N* = 477. ^*^p < 0.05, ^**^p < 0.01, ^***^p < 0.001. M, mean; SD, standard deviation; PCSA, perceived chronic social adversity; CP, cyberbullying perpetration. Gender was dummy coded with 0 for male participants and 1 for female participants.

### 3.3 Testing for mediation effect

Using the model 4 in Hayes (2013) the macro program PROCESS, mediation effects were tested after controlling for gender and age. All data were standardized. As Table 2 illustrates, PCSA had a significant total effect on cyberbullying perpetration (*β* = 0.27, *t* = 6.10, *p* < 0.001). Moreover, PCSA was significantly and positively associated with rumination (*β* = 0.20, *t* = 4.48, *p <* 0.001), rumination was significantly and positively associated with cyberbullying perpetration (*β* = 0.40, *t* = 9.74, *p* < 0.001). The indirect effect of PCSA and cyberbullying perpetration mediated by rumination was significant (*β* = 0.08, *SE* = 0.02, 95% CI = [0.04, 0.13]). The mediation effect accounted for 29.63% of the total effect. Thus, rumination partially mediated the relationship between PCSA and cyberbullying perpetration. H1 and H2 were supported.

**Table 2:** Mediation effect model.

Predictors	Model 1 (CP)			Model 2 (rumination)			Model 3 (CP)		
	*β*	*t*	95% CI	*β*	*t*	95% CI	*β*	*t*	95% CI
Gender	0.21	2.39^*^	[0.02, 0.04]	−0.07	−0.79	[−0.25, 0.11]	0.24	2.97^***^	[0.08, 0.40]
Age	0.10	1.10	[−0.08, 0.28]	0.18	1.90	[−0.01, 0.36]	0.03	0.35	[−0.14, 0.19]
PCSA	0.27	6.10^***^	[0.18, 0.35]	0.20	4.48^***^	[0.11, 0.29]	0.19	4.58^***^	[0.11, 0.27]
Rumination							0.40	9.74^***^	[0.32, 0.48]
*R*^2^	0.10			0.09			0.25		
*F*	13.61^***^			7.06^***^			32.03^***^		

^*^p < 0.05, ^**^p < 0.01, ^***^p < 0.001. PCSA, perceived chronic social adversity; CP, cyberbullying perpetration; CI, confidence interval. Each variable in the model was standardized.

### 3.4 Moderated mediation effect analysis

**Figure 2:**
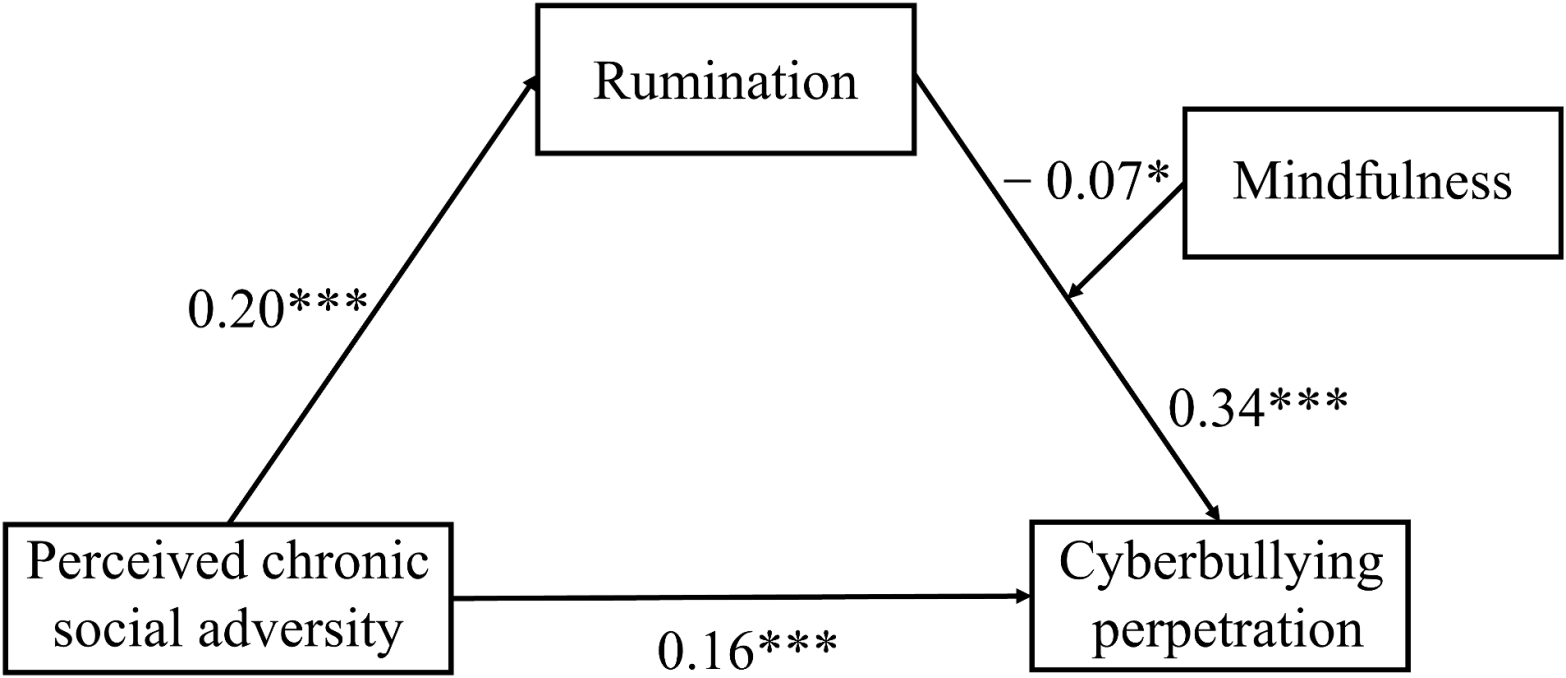
Pathway coefficients of the moderated mediation model. ^*^p < 0.05, ^**^p < 0.01, ^***^p < 0.001.

To test the moderating role of mindfulness, we ran the PROCESS macro (model 14). Moderation effects were tested after controlling for gender and age. All data were standardized. As Table 3 illustrates, the interaction of rumination and mindfulness had a significant predictive effect on cyberbullying perpetration (*β* = −0.07, *t* = −2.02, *p <* 0.05). Explain that mindfulness plays a moderating role in the prediction of rumination on cyberbullying perpetration (see Figure 2). H3 was supported.

**Table 3:** Moderated mediation model.

Predictors	Model 1 (Rumination)				Model 2(CP)			
	*β*	*SE*	*t*	95% CI	*β*	*SE*	*t*	95% CI
Gender	−0.07	0.09	−0.79	[−0.25, 0.11]	0.18	0.08	2.32^***^	[0.03, 0.33]
Age	0.18	0.09	1.90	[−0.01, 0.36]	0.03	0.08	0.36	[−0.13, 0.18]
PCSA	0.20	0.05	4.48^***^	[0.11, 0.29]	0.16	0.04	4.02^***^	[0.08, 0.24]
Mindfulness					−0.24	0.04	−5.94^***^	[−0.32, −0.16]
Rumination × Mindfulness					−0.07	0.03	−2.02^*^	[−0.14, −0.01]
Rumination					0.34	0.04	8.57^***^	[0.27, 0.42]
*R*^2^	0.06				0.32			
*F*	7.06^***^				31.29^***^			

^*^p < 0.05, ^**^p < 0.01, ^***^p < 0.001. PCSA, perceived chronic social adversity; CP, cyberbullying perpetration; SE, standard error; CI, confidence interval. Each variable in the model was standardized.

**Figure 3:**
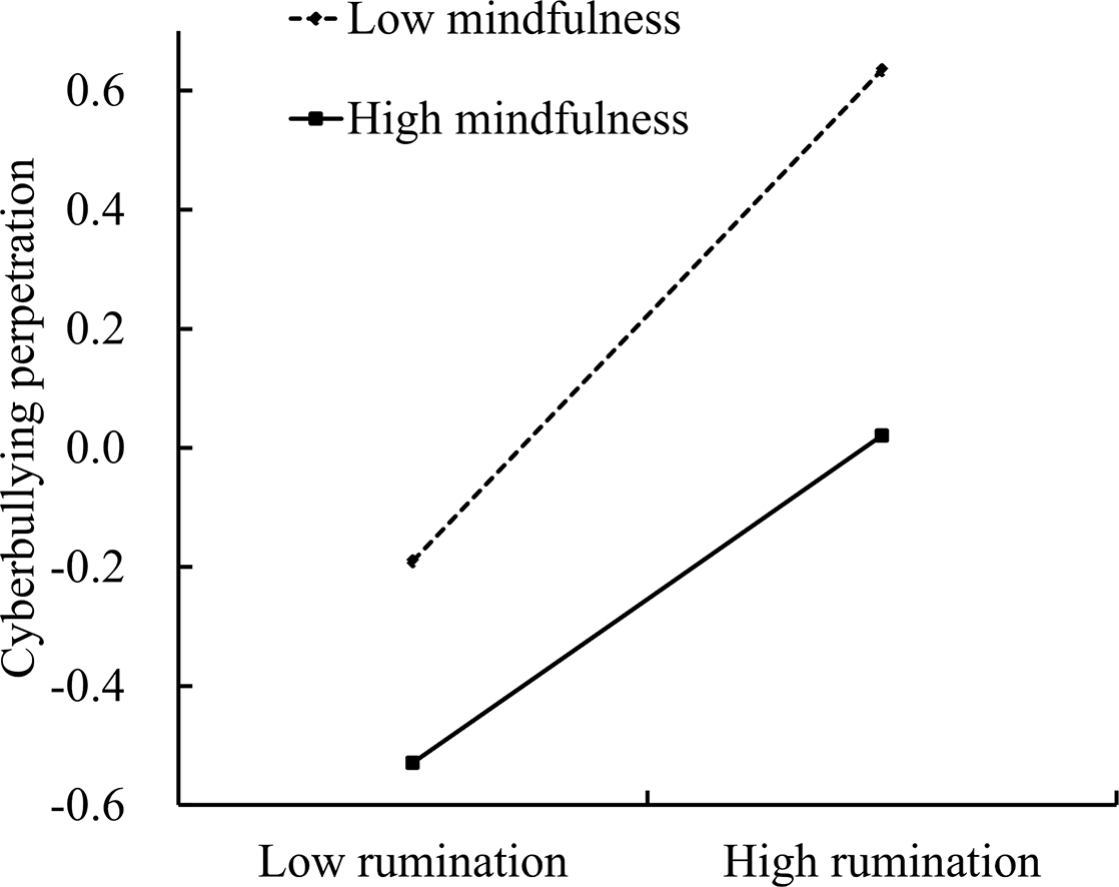
Plot of the relationship between rumination and cyberbullying perpetration at two levels of mindfulness.

To better describe the moderating effect of mindfulness, simple slope tests were conducted in this study (see Figure 3). For adolescents with lower mindfulness (*M*-1*SD*), rumination had a significant positive predictive effect on cyberbullying perpetration (*b*_simple_ = 0.41, *SE* = 0.05, *p <* 0.001, 95% CI [0.31, 0.51]). For adolescents with higher mindfulness (*M +* 1*SD*), the positive predictive effect of rumination on cyberbullying perpetration is still significant, but the predictive power is weaker (*b*_simple_ = 0.28, *SE* = 0.05, *p <* 0.001, 95% CI [0.17, 0.38]). It showed that as mindfulness increases, the predictive effect of rumination on cyberbullying perpetration is weakened. This suggested that mindfulness can alleviate the negative impact of rumination on cyberbullying perpetration (see Table 4).

**Table 4:** The conditional indirect effects of mindfulness.

Mindfulness	Effect	Boot *SE*	Boot LLCI	Boot ULCI
M − 1SD	0.08	0.02	0.04	0.13
M	0.07	0.02	0.03	0.12
M + 1SD	0.06	0.02	0.02	0.11

SE, standard error; CI, confidence interval; LL, lower limit; UL, upper limit.

## 4 Discussion

In current mobile Internet era, an increasing number of adolescents are walking on the edge of cyberbullying perpetration (Chu et al., 2019; Fan et al., 2019). Although the antecedents and potential adverse consequences of cyberbullying perpetration have garnered considerable empirical support, much less is known about whether and how subjective perception of negative life events potentially increases in adolescents’ cyberbullying perpetration. Thus, we formulated and examined a moderated mediation model based on previous empirical studies and theories to clarify not only the association between PCSA and cyberbullying perpetration among adolescents, but also reveal the mediating role of rumination and the moderating role of mindfulness. Our findings have both theoretical and practical significance, as they enhance the understanding of the connection between PCSA and cyberbullying perpetration and provide empirical support for designing intervention strategies from the perspectives of cognitive strategies, emotional reactions, and social relationships.

Regarding age differences, we found a negative correlation between age and PCSA. The reason could be that first-grade students have just entered a new environment and are just beginning to develop their capabilities to adapt to stressors in high school life. Instead, second-grade students have already gained trustworthy and supportive interpersonal relationships. During several months of study, second-grade students are also becoming more mature in academic competition. The result suggests that becoming older implies that an individual will have more experience in coping with negative life events (Mann et al., 2015), so older age can become a protective factor for adolescent (Zhang J et al., 2017). However, no age differences in rumination, mindfulness, and cyberbullying perpetration were found in this study. Because the sampling age range in the study is narrow and these participants are in the same intrinsic cultural context and a similar developmental stage in maladaptive cognitive strategy and emotion regulation (Zhang et al., 2022). Moreover, they are also in the age group associated with greater access to new internet use and almost the highest cyberbullying perpetration rate (López-Castro et al., 2023).

Regarding gender differences, we found females have higher PCSA than males. The reason may be that females were more vulnerable and more often reported a strong negative impact on psychological well-being when they encountered social adversity (Seematter-Bagnoud et al., 2010; Lu et al., 2020). So, once negative life events happen to them, females are more likely to have psychological distress than males. The results showed that males scored higher than females in mindfulness, which was in agreement with Yang XJ et al. (2021). One explanation may be that males have a greater tendency towards present hedonism than females (Marks et al., 2010; Fuentes et al., 2022). However, no gender differences in rumination and cyberbullying perpetration were found in this study. Some researchers reviewed the potential influences of developmental stages on gender differences in rumination (Johnson and Whisman, 2013). Compared with adults, the gender differences in rumination are quite small or even not significant among adolescents. The reason may be that adolescents are not gender specific and generally lack methods of coping in response to a broad range of stressors (Dickson et al., 2017). In addition, gender differences may not always be as prominent in cyberbullying perpetration (Barlett and Coyne, 2014). Considering the prevalent use of the internet in the lives of adolescents and the properties of cyberbullying, this may be because gender differences in cyberbullying appear to be driven by discrepancies in multiple male–female motivational factors, such as cognitive empathy and perceived online disinhibition (Wong et al., 2018).

### 4.1 The relationship between PCSA and cyberbullying perpetration

The total effect model demonstrates that PCSA significantly and positively associated with cyberbullying perpetration among adolescents. Adolescents with higher PCSA are more inclined to bully others in cyberspace, thus validating H1. Support the general strain theory and previous research findings (Huang et al., 2017; Ding et al., 2018).

Adolescents who have higher PCSA have accumulated a large amount of aggression-related clues (such as social exclusion and frustration, etc.) through long-term interaction with negative life events, which increase their susceptibility to social adversities (Zhang J et al., 2017; Davis et al., 2018; Gabbiadini and Riva, 2018). They will automatically implement an immediate assessment of the social adversity when they encounter certain clues. This assessment process is spontaneous and unconscious and requires almost no cognitive effort. Adolescents can directly and automatically engage in bullying perpetration and aggression based on the original cognitive scripts when they think they face threatening events in their daily lives (Anderson and Bushman, 2002; Allen et al., 2018; Wang et al., 2019). Additionally, adolescents with heightened PCSA are prone to feel frustrated in reality and form latent negative self-schemas, resulting in a negative affect state for a long time, which also may enhance their motivation to implement aggressive behaviors in cyberspace to rebuild confidence (Ding et al., 2018). Particularly in cyberspace with its unique characteristics including anonymity and accessibility, the activation level of the adolescents’ behavioral inhibition system is lower than that in reality (Yen et al., 2012). Therefore, adolescents with higher PCSA are more likely to learn that bullying others in cyberspace is an acceptable way to alleviate their chronic negative effects and escape from negative life events in real social interaction (Zhang et al., 2019; Yuan et al., 2020). Previous studies found that PCSA can lead to a series of offline maladaptive problems including social avoidance, self-injury, and suicidal tendencies (Ding et al., 2018; Liu et al., 2022). Our findings extend existing research, indicating that adolescents with high levels of PCSA may not only become victims in reality but also be more likely to bully others in cyberspace.

### 4.2 Rumination as a mediator

This study’s results indicate that PCSA is associated with cyberbullying perpetration through the partial mediation of rumination, thereby validating H2. This finding supports the general aggressive model (Anderson and Bushman, 2002). PCSA may be a necessary condition in the formation of cyberbullying perpetration as a distal contributory, while rumination may be a sufficient condition for the formation of cyberbullying perpetration as a proximal contributory. Therefore, PCSA is not only directly associated with cyberbullying perpetration, but also indirectly associated with cyberbullying perpetration through rumination. This finding also supports the views of the social information processing theory that individuals’ perception of chronic social adversity may be a triggering factor for cyberbullying perpetration, and individuals will engage in emotional processing of the triggering factor, promoting the expression of cyberbullying perpetration (Crick and Dodge, 1994).

On the one hand, this study’s results are consistent with previous findings that a positive association exists between subjective perceptions of negative life events and rumination (Michl et al., 2013; Liu and Wang, 2017; Jin et al., 2020). Adolescents with high PCSA are often most sensitive to negative events, so they have to constantly and repeatedly perceive social adversity information in their daily lives (Ding et al., 2018). However, their cognitive and emotional management abilities are not yet mature, and they have not formed an appropriate cognitive style, which makes it difficult for them to form reasonable assessments of such events (Dickson et al., 2017). They have to repeatedly try to integrate the social adversity information into new life narratives to reduce the psychological pain caused by it (Platte et al., 2022). Therefore, high PCSA may encourage adolescents to develop non-adaptive passive coping styles like rumination into negative psychological habits related to emotional reactions, thus causing incorrect cognitive style tendencies, inducing excessive negative emotional experiences, and repeatedly thinking about possible strategies and consequences (Zhang J et al., 2017; Jin et al., 2020).

On the other hand, the results are consistent with previous studies that have shown that individuals with higher rumination are more likely to exhibit bullying perpetration and aggression (Pedersen et al., 2011; Ruddle et al., 2017; Jin et al., 2020). In online contexts, Yang J et al. (2021) also found a significant positive correlation between adolescents’ anger rumination and cyberbullying perpetration. Adolescents with higher rumination are more likely to immerse in the perception of social adversity, this will reduce their critical value of the potential threat evaluation system (Mathews and MacLeod, 2002), resulting in their abnormal sensitivity to negative events and difficulty to shift their attention from the negative emotions and experiences, ultimately leading to the deviation of cognition, the reduction of empathy, the formation of hostile prejudice, and the phenomenon of “violent desensitization” (Wang et al., 2018). That is to say, rumination reduces the self-regulation ability of adolescents when facing social adversity, and they are more likely to bully others in cyberspace (Gao et al., 2021). As such, adolescents’ subjective perceptions of negative life events affect their cyberbullying perpetration by exacerbating their rumination.

### 4.3 Mindfulness as a moderator

Further, this study discovered that the mediating effect of rumination was moderated by mindfulness. Specifically, the positive association between rumination and cyberbullying perpetration among adolescents was progressively weakened as mindfulness levels increased, confirming H3. These findings align with the buffering hypothesis within the “delivering carbon in the snow model,” which suggests that a protective factor may diminish the predictive effect of a risk factor on the outcome variable (Zhang et al., 2022). In this study, mindfulness served as a protective factor and rumination served as a risk factor for cyberbullying perpetration among adolescents. These two adverse factors interacted, which had an impact on the outcomes, with mindfulness effectively buffering the association between rumination and cyberbullying perpetration and aggression.

This may be because, in the limited resource of self-control theory (Baumeister et al., 2007), individuals with higher mindfulness will accumulate self-control resources and emphasize the subjective attitude of acceptance and non-judgment, as well as the process of “re-perception” towards external stimuli, including negative life events (Zou et al., 2021). They will facilitate their capacity to receptively observe negative life events as they arise with acceptance and equanimity through mindfulness, and make flexible rather than automated responses to those events objectively, which in turn buffer initial threat appraisals and increase secondary appraisals of coping resources (Creswell and Lindsay, 2014). Specifically, adolescents with higher mindfulness usually have higher self-efficacy, psychological resilience, and other positive psychological resources (Yue et al., 2020; Xiong et al., 2023), which can effectively alleviate the insufficient self-control resources caused by rumination and enhance individuals’ abilities to execute self-control, reduce repetitive passive thinking about adverse events, and promote individuals to seek more proactive strategies such as self-withdrawal (Petrova et al., 2021). Therefore, mindfulness enables adolescents facing social adversity to consciously choose and recognize their thoughts, emotions, and feelings but does not generate habitual reactions, then gradually eliminating the automated evaluation process of distressed emotions (Chambers et al., 2009), thus reducing the likelihood of engaging in deviant behaviors in cyberspace. Just as the superego in psychoanalytic theory strengthens its control over the id, it avoids the implementation of socially unacceptable behaviors that result from attempts to vent negative emotions (Chen et al., 2022).

Previous research emphasized that mindfulness includes two components: control of attention and guidance of individual experience (Duan, 2014). Individuals with low mindfulness might find it difficult to pay attention to the awareness of current cognition, thinking, and behavior, so they may lack confidence in their capacity to interpret challenging events or stimuli as new experiences and then have to repeatedly judge them, thereby amplifying their hostile reactions to the outside world and themselves (Borders et al., 2010). Conversely, individuals with higher mindfulness possess a more objective evaluation of capabilities (Creswell and Lindsay, 2014). They gain a stronger sense of control over their attention resources (Kraemer et al., 2016). Hence, they are more capable of shifting the resources from self-reflection to exploration of new experiences, which can alleviate their negative self-evaluation and negative emotions caused by rumination, promote their stable emotional states, and enhance their monitoring and restraint of their behaviors, then alleviate the risk of cyberbullying perpetration (Kowalski et al., 2014). Due to the constraints of adolescents’ social experience, those with low mindfulness will face great challenges in their tolerance and acceptance of negative life events (Xiong et al., 2023). Mindfulness helps adolescents to assess their inner thoughts and external environment as comprehensively as possible, without being limited by their own experiences when facing social adversity (Zhang et al., 2022). Therefore, a mindfulness-based intervention would be helpful to enhance the self-control function of adolescents with high PCSA, they can be more inclined to adjust cognitive strategies to buffer negative emotional experiences caused by rumination and then reduce the risk of cyberbullying perpetration.

### 4.4 Implications and limitations

This study revealed the association between PCSA and cyberbullying perpetration as well as the mechanism in between, which added to our understanding of the explored constructs and brought practical implications. From a theoretical perspective, the results provide further validation for the general strain theory in the context of cyberbullying perpetration by revealing a significant positive correlation between PCSA and cyberbullying perpetration among adolescents. Additionally, few studies have investigated cyberbullying perpetration from the perspective of the interaction of multiple factors (Fan et al., 2018). This study provided an attempt to explore the mechanism of cyberbullying perpetration more systematically and comprehensively by focusing on the perspectives of cognitive strategies, emotional reactions, and social relationships, and further revealed the mediating role of rumination between PCSA and cyberbullying perpetration. The results verified the applicability of the general aggressive model in cyberbullying situations. Furthermore, previous studies have mostly used environmental variables such as negative life events as independent variables to explore the effects on individuals. Our findings explored the issue of individual consistency of the effect caused by subjective perception of negative life events from a new perspective. The moderated mediation model examined in this study revealed the mechanisms of perceived chronic social adversity, rumination, and mindfulness on aggressive behaviors in cyberbullying situations, which not only validated the limited resource of self-control theory in the context of cyberbullying among adolescents, but also explored whether perceptions of social adversity have the same impact on individuals with different levels of mindfulness, which is beneficial for further revealing the interaction between mindfulness and subjective perception of negative life events on individuals, thus supplied new insights for preventing and intervening cyberbullying perpetration among adolescents.

From a practical standpoint, the results offer guidance on promoting mindfulness-based intervention through educational measures in response to cyberbullying incidents and present new perspectives for educators to reduce the prevalence and negative impact of cyberbullying from the perpetrators. To prevent adolescents from experiencing long-term psychological pain and accumulating the risk of cyberbullying, educators should help adolescents improve their perception abilities to integrate various types of information in life, correct negative cognitive biases, and seek a positive meaning from adversity based on their advantages. For instance, conducting training programs in social skills and team games to enhance adolescents’ adaptive social information processing, emotional response, and social skills, then guide them to establish reasonable expectations for social adversity. Assisting adolescents to explore their unique feelings of exclusion and rejection in multiple identities and social environments, helping them to conduct other supportive relationships and areas of achievement, and executing principles of dialectical-behavioral therapy like establishing core skills in the regulation of emotional responses, distress tolerance, and interpersonal efficacy. Moreover, this study revealed that educators should pay prompt attention to the perceived chronic social adversity and rumination of adolescents, and assist them in cognitive adjustment through mindfulness-based intervention. For instance, Mindfulness-Based-Cognitive Therapy (MBCT) is a systemic strategy that combines elements of cognitive behavioral therapy and mindfulness skills, which can help adolescents accept negative events with a more open mindset. MBCT can also make individuals aware of the possibility of increasing their flexibility in response when they are about to fall into habitual rumination, then reducing rumination and promoting positive mental health (Proeve et al., 2018).

This study had several limitations. Firstly, all variables were measured by self-reported methods, so the objective details of social adversities received little attention, which may be subject to social desirability bias and lack of ecological validity. Future studies could employ a unified standardized stimulus to evaluate the response and emotional arousal level of adolescents to adversities, which could improve the validity of data. For instance, standardized negative life events (presented by scenarios or vignettes that describe common experiences and feelings of adolescents who implied such events) could be used as stimuli to gather the adolescents’ reports of their emotions and feelings about social adversities in strictly controlled situational experiments, so that we would be more confident that we were assessing change in responses related to social adversities rather than change in stressors. This method could improve validity and accuracy over retrospective recall of actual events and reactions and has been valuable as a commonly used approach to assessing responses to stress among large samples in other research areas (Catterson and Hunter, 2010; Zimmer-Gembeck et al., 2011). Secondly, the cross-sectional data could not permit the causality portrayed by the existing theoretical models. Longitudinal experimental design could be applied to explore the direction and the long-term impact between variables. For instance, the use of the ecological momentary assessment method may be more effective in capturing the fluctuation patterns of participants’ cognitive emotional reactions and rumination under different clues such as negative or ambiguous stimuli in social situations, even evaluating the negative emotion and attention allocation of participants when facing different levels of potential threats, thus revealing the dynamic impact of PCSA on cyberbullying perpetration. Thirdly, this study investigated the degree of subjective perception of negative life events among adolescents but did not specifically explore the role of early cyber victimization experience in the formation of cyberbullying perpetration. Compared to other negative life events, as a typical adverse event in cyberbullying scenarios, cyber victimization is more likely to lead to cyberbullying, thus creating a vicious cycle (Sun et al., 2020). Prior literature has found a strong correlation between cyber victimization and cyberbullying perpetration, but the underlying mechanisms remain unclear (Luo et al., 2023). Future studies could further explore the association between early cyber victimization experience and cyberbullying perpetration to examine the mechanisms underlying the role transition from victim to bully in cyberbullying incident. Lastly, the hypothesis mainly focused on rumination that produces negative effects, but rumination includes multiple dimensions, such as maladaptive dimensions of negative passivity and adaptive dimensions of positive problem-solving (Watkins, 2008; Pearson et al., 2011). Future studies could further investigate the interaction between PCSA and different dimensions of rumination to examine their distinct roles in various emotional regulation strategies and aggressive behaviors.

## 5 Conclusion

This study investigated the association between PCSA and cyberbullying perpetration, and explored the mediating role of rumination and the moderating role of mindfulness. Our findings indicated that PCSA is significantly and positively associated with cyberbullying perpetration among adolescents, and rumination partially mediates the relationship between PCSA and cyberbullying perpetration. Furthermore, mindfulness moderated the association between rumination and cyberbullying perpetration. In particular, the association between rumination and cyberbullying perpetration is greater for adolescents with lower mindfulness compared to those with higher mindfulness.

## Data availability statement

The raw data supporting the conclusions of this article will be made available by the authors, without undue reservation.

## Ethics statement

The studies involving humans were approved by the Research Ethics Committee of Central China Normal University. The studies were conducted in accordance with the local legislation and institutional requirements. Written informed consent for participation in this study was provided by the participants’ legal guardians/next of kin.

## Author contributions

RC: Methodology, Writing – review & editing, Conceptualization. YH: Writing – review & editing. H-fC: Writing – review & editing. YF: Writing – review & editing. C-yF: Writing – review & editing, Supervision, Project administration, Funding acquisition.
